# Metabolic Tumour Volume from PSMA PET/CT Scans of Prostate Cancer Patients during Chemotherapy—Do Different Software Solutions Deliver Comparable Results?

**DOI:** 10.3390/jcm9051390

**Published:** 2020-05-08

**Authors:** Philipp E. Hartrampf, Marieke Heinrich, Anna Katharina Seitz, Joachim Brumberg, Ioannis Sokolakis, Charis Kalogirou, Andreas Schirbel, Hubert Kübler, Andreas K. Buck, Constantin Lapa, Markus Krebs

**Affiliations:** 1 Department of Nuclear Medicine, University Hospital Würzburg, 97080 Würzburg, Germany; mariekeheinrichmh@gmail.com (M.H.); brumberg_j@ukw.de (J.B.); schirbel_a@ukw.de (A.S.); buck_a@ukw.de (A.K.B.); constantin.lapa@uk-augsburg.de (C.L.); 2 Department of Urology and Paediatric Urology, University Hospital Würzburg, 97080 Würzburg, Germany; seitz_a3@ukw.de (A.K.S.); Kalogirou_c@ukw.de (C.K.); kuebler_h@ukw.de (H.K.); krebs_m@ukw.de (M.K.); 3 Department of Urology, Martha-Maria Hospital Nuremberg, 90491 Nuremberg, Germany; ioannis.sokolakis@martha-maria.de; 4 Nuclear Medicine, Medical Faculty, University of Augsburg, Stenglinstrasse 2, 86156 Augsburg, Germany; 5 Comprehensive Cancer Center Mainfranken, University Hospital Würzburg, 97080 Würzburg, Germany

**Keywords:** prostate-specific membrane antigen (PSMA), metabolic tumour volume (MTV), total lesion PSMA, biomarker, software, comparability, agreement

## Abstract

(1) Background: Prostate-specific membrane antigen (PSMA)-derived tumour volume (PSMA-TV) and total lesion PSMA (TL-PSMA) from PSMA PET/CT scans are promising biomarkers for assessing treatment response in prostate cancer (PCa). Currently, it is unclear whether different software tools for assessing PSMA-TV and TL-PSMA produce comparable results. (2) Methods: ^68^Ga-PSMA PET/CT scans from *n* = 21 patients with castration-resistant PCa (CRPC) receiving chemotherapy were identified from our single-centre database. PSMA-TV and TL-PSMA were calculated with Syngo.via (Siemens) as well as the freely available Beth Israel plugin for FIJI (Fiji Is Just ImageJ) before and after chemotherapy. While statistical comparability was illustrated and quantified via Bland-Altman diagrams, the clinical agreement was estimated by matching PSMA-TV, TL-PSMA and relative changes of both variables during chemotherapy with changes in serum PSA (ΔPSA) and PERCIST (Positron Emission Response Criteria in Solid Tumors). (3) Results: Comparing absolute PSMA-TV and TL-PSMA as well as Bland–Altman plotting revealed a good statistical comparability of both software algorithms. For clinical agreement, classifying therapy response did not differ between PSMA-TV and TL-PSMA for both software solutions and showed highly positive correlations with BR. (4) Conclusions: due to the high levels of statistical and clinical agreement in our CRPC patient cohort undergoing taxane chemotherapy, comparing PSMA-TV and TL-PSMA determined by Syngo.via and FIJI appears feasible.

## 1. Introduction

Imaging-derived biomarkers and clinical response classification systems such as RECIST 1.1 [1] for CT scans and PERCIST 1.0 for PET/CT scans [2] play a crucial role in cancer care. Among potential biomarkers, metabolic tumour volume seems to be especially promising for clinicians as it represents a measure for the whole-body tumour burden. This principal attractiveness has led to several studies examining metabolic tumour volume and total lesion values for various solid and haematological malignancies, e.g., ^18^F-FDG PET/CT scans and their prognostic impact for tumour entities such as cervical cancer [3], head and neck [4,5], pancreatic cancer [6], Hodgkin’s lymphoma [7], ovarian [8] and prostate cancer (PCa) [9].

Given the essential role of PSMA PET/CT-based diagnostics in modern PCa care, the potential predictive value of whole-body PSMA tumour volume (PSMA-TV) and whole-body total lesion PSMA (TL-PSMA) derived from PSMA PET/CT scans has been examined [10].

However, a variety of software algorithms delivers metabolic tumour volume markers such as PSMA-TV and TL-PSMA. Accordingly, studies examining metabolic tumour volume derived from PSMA PET/CT used different software tools [11,12,13,14].

For researchers, it is currently unclear whether results obtained by different programs are comparable between different centres, e.g., in large multicentre studies. Likewise, comparability of data on tumour volume is critical when patients change the diagnostic department and are rated with a different software tool. In general, for prospective biomarkers in clinical use, it should be clear whether choice of the calculation tool influences results. To our knowledge, there are no existing data on these topics for PSMA-TV and TL-PSMA.

For this reason, we calculated PSMA-TV and TL-PSMA with Syngo.via and the Beth Israel plugin for FIJI (Fiji Is Just ImageJ, open source project) from PSMA PET/CTs of castration resistant prostate cancer (CRPC) patients, representing a cohort of late stage disease patients with a high tumour burden. Moreover, we assessed dynamics induced by chemotherapy (docetaxel or cabazitaxel) by determining PSMA-TV and TL-PSMA with both algorithms at two time points – before and after completion or termination of chemotherapy. We analyzed statistical comparability of both software tools using Bland–Altmann plotting. Clinical agreement was estimated by matching PSMA-TV, TL-PSMA and relative changes of both variables during chemotherapy with changes in serum PSA levels (ΔPSA) and PERCIST (Positron Emission Response Criteria in Solid Tumors).

## 2. Materials and Methods

### 2.1. Study Cohort

We searched our database of *n* = 1025 patients who received a PSMA PET/CT between July 2014 and December 2018 at our centre and identified a clinically distinct subgroup of *n* = 21 patients receiving docetaxel or cabazitaxel as systemic chemotherapy for metastatic PCa in a castration-resistant stage (CRPC). All patients included had at least one ^68^Ga-PSMA PET/CT in a three months period before chemotherapy (in the following named baseline) and another scan in a four months period after completion or termination of chemotherapy (follow-up). Serum prostate-specific antigen (PSA) levels were determined at baseline and after completion or termination of chemotherapy. Table 1 further outlines the characteristics of our study cohort.

All findings, data acquisition and processing in this study comply with the ethical standards laid down in the latest Declaration of Helsinki as well as with the statutes of the Ethics Committee of the University of Würzburg concerning anonymized retrospective medical studies. There were no fundamental ethical and legal objections to the evaluation of the listed data (# 20,191,106 02 from November 2019).

### 2.2. PSMA PET/CT Imaging Protocol

Images were obtained with ^68^Ga-PSMA I&T (^68^Ga-PSMA) which was prepared using a cassette-based radiotracer synthesis module (Scintomics, Fürstenfeldbruck, Germany). Briefly, the eluate (^68^Ga^3+^ in 0.1 M HCl) of a ^68^Ge/^68^Ga-generator (GalliaPharm, Eckert & Ziegler AG, Berlin, Germany) was transferred to a cation exchange cartridge, eluted, added to a solution of 20 µg PSMA I&T (Scintomics, Fürstenfeldbruck, Germany) in HEPES (4-(2-hydroxyethyl)-1-piperazineethanesulfonic acid)-buffer and heated. The product was immobilized on a SepPak C18-cartridge, washed with water und eluted with ethanol/water. The final eluate was passed through a sterile filter into a sterile vial und diluted with phosphate buffer solution. Variations in injected radiotracer activity were caused by the short half-life of ^68^Ga and variable elution efficiencies obtained during the lifetime of the ^68^Ge/^68^Ga radionuclide generator.

All patients received a diluted oral contrast agent (Peritrast CT 400 mg iodine/mL). Furosemide 10mg was administered at the time point of tracer injection. Patients underwent ^68^Ga-PSMA PET/CT from the skull base to the mid-thigh using a Biograph mCT scanner (Siemens Medical Solutions, Erlangen, Germany) after an average incubation time of 71 min (interquartile range (IQR) 54–82 min; baseline)/82 min (IQR 64–84 min; follow-up). A mean of 119 MBq ^68^Ga-PSMA (IQR 108–132 MBq; baseline)/125 MBq (IQR 105–144 MBq; follow-up) was injected per patient. PET/CT included a diagnostic CT scan (mA modulated, 120kV, 5 mm slice thickness) in the portal venous phase (70 s after injection of Imeron 350 at 1 mL/kg body weight). All PET scans were acquired in three-dimensional mode with an acquisition time of 2 min per bed position. Images were reconstructed iteratively using an ordered subset expectation maximization algorithm (3 iterations, 24 subsets) followed by a post-reconstruction Gaussian filter smoothing (2 mm full-width at half-maximum; Siemens TrueX, Siemens Healthineers, Erlangen, Germany).

### 2.3. Image Analysis

First, PET/CT images were analysed using a dedicated workstation equipped with a commercial software package (Syngo.via; VB30A, Siemens Healthcare, Erlangen, Germany). For comparison of the derived parameters, we then used the Beth Israel plugin for FIJI [15], a freely available shareware from the Beth Israel Deaconess Medical Center (Boston, MA, USA), Division of Nuclear Medicine and Molecular Imaging.

All lesions with visually higher uptake compared to environment were rated as PSMA-positive, suggesting local recurrence or metastases. Structures with known non-malignant uptake of PSMA (e.g., salivary glands and coeliac ganglia) were excluded. Diagnostic decisions were made by consensus of two experienced nuclear medicine physicians (A.K.B., C.L.) and one radiologist. For all suspected pathological lesions, mean and maximum standardized uptake value (SUVmean, SUVmax) and the metabolic tumour volume were determined with two different software applications (Syngo.via and the Beth Israel plugin for FIJI—in the following named FIJI). In addition, we determined the hottest lesion (highest SUVmax of all lesions) for each patient. 

In both software applications, we used a semi-automatic analysis (Syngo.via with volumes of interest (VOIs) with isocontours; FIJI with the automatic segmentation function as described by the developers). For both applications, the threshold was set by a 3 cm spherical region of interest (ROI) in the liver adapted from PERCIST and PROMISE criteria (threshold: 1.5 × liver mean + 2 × standard deviation) [2,16]. In case of liver metastases, we set a threshold based on a 1 cm diameter ROI in descending thoracic aorta extended over 2 cm *z*-axis (threshold: 2 × aortic mean + 2 × standard deviation). After automated analysis, a lesion-based manual control was carried out by two researchers (P.E.H., M.H.). Decisions were made by consensus. From these parameters, the PSMA uptake of each lesion was calculated by multiplying the respective metabolic tumour volume with SUVmean [10]. Summing up all lesions revealed PSMA-TV and TL-PSMA for each patient.

### 2.4. Clinical Response Criteria

We calculated changes in serum PSA levels in order to determine biochemical response (BR). Partial response (PR), stable disease (SD) and progressive disease (PD) were used as defined previously [17]. As summarized in Table 2, we applied the following clinical endpoint criteria. Relative changes in serum PSA levels, SUVmax (PERCIST criteria) as well as PSMA-TV and TL-PSMA were determined by comparing values at the time point of follow-up and baseline (rel. ΔX [%] = X_follow-up_/X_baseline_ × 100). We arbitrarily set a relative decrease or a relative increase of 30% as threshold for PR and PD (see Table 2). 

To assess PET/CT-derived response, we used the PERCIST 1.0 criteria and adapted them to PSMA PET/CT [2]. The lesion with the highest SUVmax was determined for both PET/CT studies regardless of the overall number of lesions.

### 2.5. Statistical Analysis

We performed statistical analyses with SPSS software (Version 25, IBM Corp., Armonk, NY, USA) and applied Shapiro-Wilk tests for normal distribution. Due to the non-normal distribution of our datasets at baseline and follow-up, we used median and interquartile range (IQR) for further characterization. We then tested for significant differences between non-normally distributed datasets by applying Wilcoxon rank tests. For correlation analyses, we used the Spearman rank correlation coefficient.

We performed a power analysis by applying the G*Power tool [18]. There were no existing preliminary data regarding differences between software solutions. However, as implied by our arbitrarily set clinical response criteria (±30% changes in PSMA-TV and TL-PSMA; see Section 2.4.), we wanted to rule out that clinically relevant differences between the two software solutions were missed within our study. Therefore, effect size of the difference was considered large and consecutively set at 0.7. For a power of 80% and α = 0.05, the required sample size was *n* = 19.

Figures—including Bland-Altman plots—were generated with PRISM (Graphpad Software, San Diego, CA, USA). In specific, Bland–Altman diagrams [19] were used for assessing the statistical agreement between values derived from both software algorithms, Syngo.via and FIJI. Instead of absolute differences, we examined relative differences between both algorithms within Bland–Altman plotting. Dotted lines in the plots represented the limits of agreement of both applications for each timepoint and each biomarker candidate—assuming a reasonable statistical comparability of both software solutions within these boundaries. Repeatability coefficients (RC) were calculated from the same dataset as described previously [20]: RC = 1.96 × standard deviation of relative differences. 

## 3. Results

We identified *n* = 21 PCa patients from our single-centre ^68^Ga-PSMA PET/CT database undergoing docetaxel or cabazitaxel chemotherapy as systemic treatment for CRPC. Each patient had at least one baseline PET/CT scan and one follow-up scan after completion or termination of chemotherapy. 

### 3.1. PSMA-TV and TL-PSMA during Chemotherapy—Determined by Syngo.via and FIJI

**Figure 1:**
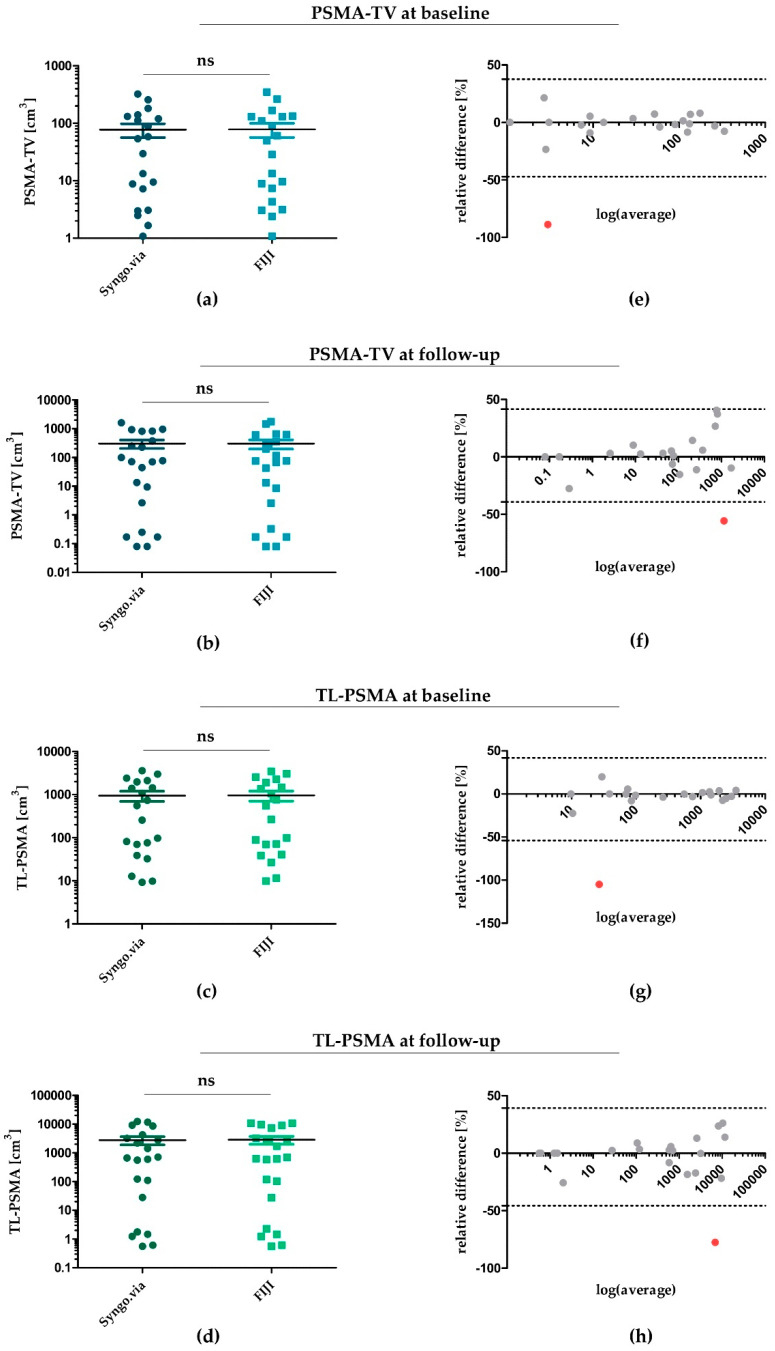
Statistical agreement of PSMA-TV and TL-PSMA determined by Syngo.via and FIJI at baseline and follow-up. (**a**,**b**) PSMA-TV for each patient at baseline (**a**) and follow-up (**b**)—determined by Syngo.via and FIJI. (**c**,**d**) TL-PSMA at baseline (**c**) and follow-up (**d**) determined by both software algorithms. (**e**–**h**) Bland–Altman diagrams for illustration and assessment of statistical comparability for each patient. Single outliers with a significant divergence between values determined by Syngo.via and FIJI were marked in red—all others were depicted within a range of 1.96 × standard deviation (SD). PSMA: prostate-specific membrane antigen; PSMA-TV: PSMA tumour volume; TL-PSMA: total lesion PSMA; ns: not significant.

In a first step, we determined PSMA-TV and TL-PSMA for each patient from baseline and follow-up PET/CT scans by applying both, the Syngo.via and the FIJI software. Figure 1 illustrates respective results (raw data for each patient are available in Supplementary Table S1).

Regarding PSMA-TV at baseline (Figure 1a) and follow-up (Figure 1b), applying Syngo.via revealed a median value of 41.7 cm^3^ (IQR: 124.3 cm^3^) at baseline and a median of 70.8 cm^3^ (IQR: 598.2 cm^3^) at follow-up. Applying the FIJI software yielded comparable PSMA-TV values for both time points—resulting in a median of 39.3 cm^3^ (IQR: 125.4 cm^3^) for baseline (*p* = 0.48) and a median of 75.1 cm^3^ (IQR: 485.4 cm^3^) for follow-up PET/CT scans (*p* = 0.43). Correspondingly, TL-PSMA determined by FIJI was also comparable at baseline and follow-up (Figure 1c,d). While applying Syngo.via yielded a median of 406.3 cm^3^ (IQR: 1794.4 cm^3^) and 663.39 cm^3^ (IQR: 3687.7 cm^3^) for baseline and follow-up scans, using FIJI led to a median of 411.6 cm^3^ (IQR: 1742.8 cm^3^) (*p* = 0.39) and 626.3 cm^3^ (IQR: 5196.7 cm^3^) (*p* = 0.71), respectively.

To assess statistical agreement of Syngo.via- and FIJI-determined PSMA-TV and TL-PSMA, we next plotted Bland-Altman diagrams for PSMA-TV and TL-PSMA at baseline and follow-up (Figure 1e–h). In line with PSMA-TV and TL-PSMA values illustrated in Figure 1a–d, Bland–Altman diagrams confirmed a good statistical comparability of both software algorithms—with 20 of 21 measurements being within the calculated limits of statistical agreement (1.96 × standard deviation of relative differences; dotted lines in Figure 1e–h) at each time point. Only one data point within each plot was located outside the boundaries of statistical agreement (indicated by the dotted lines)—with relative differences of both software tools indicated on the y axis. These outliers (marked in red) represented measurements of only two patients from our study cohort: patient #5 for baseline values of PSMA-TV (Figure 1e) and TL-PSMA (Figure 1g) and patient #11 for follow-up values of PSMA-TV (Figure 1f) and TL-PSMA (Figure 1h).

When calculating repeatability coefficients (RC), PSMA-TV at follow-up (Figure 1f) revealed the lowest RC of 40.4%, followed by PSMA-TV at baseline with 42.4% (Figure 1e), TL-PSMA at follow-up with 41.5% (Figure 1h) and TL-PSMA at baseline with 48.0% (Figure 1g).

### 3.2. PSMA-TV and TL-PSMA as Imaging-Derived Biomarkers in CRPC

To further assess the clinical agreement of metabolic tumour volume determined with different software solutions, we calculated correlation of PSMA-TV and TL-PSMA with serum PSA levels. Table 3 illustrates our results. We found highest correlation for follow-up data—with ρ = 0.86 reaching its maximum for PSMA-TV determined by FIJI and serum PSA at time of follow-up. Regarding relative changes, application of FIJI yielded comparable correlations for ΔPSMA-TV and ΔTL-PSMA with ΔPSA (ρ = 0.67 and ρ = 0.69, respectively) compared to Syngo.via (ρ = 0.70 and ρ = 0.67, respectively). In summary, applying Syngo.via and FIJI delivered comparable and significantly positive correlation coefficients for PSMA-TV/TL-PSMA and serum PSA.

In a next step, we stratified all CRPC patients in CR, PR, SD and PD subgroups—depending on ΔPSMA-TV and ΔTL-PSMA. As already described in Table 2, we used an arbitrarily set threshold of ±30% change. Table 4 shows patients’ classifications according to the Syngo.via and the FIJI software as well as the established clinical endpoints PERCIST and BR. 

Of note, stratifying patients according to ΔPSMA-TV and ΔTL-PSMA response criteria yielded identical response classifications for each patient—independent of the software algorithm.

To learn more about the prognostic potential of ΔPSMA-TV and ΔTL-PSMA in our small study cohort, we patient-wise compared our therapy response subgroups based on metabolic tumour volume with the clinically established endpoints BR and PERCIST. Regarding BR, five patients showed a PR, three patients were classified as SD and eleven patients as PD. Two patients (#2 and #17) had very low PSA values, which had not reflected treatment responses in previous therapy lines. For this reason, they were marked as PSA-negative PCa by responsible urologists. Accordingly, we did not perform BR classification. 

Response assessment based on PERCIST criteria showed four patients with PR, one patient with SD and sixteen patients with PD. Ten patients were rated PD due to new lesions and not due to rising SUVmax. Calculating ΔPSMA-TV and ΔTL-PSMA with Syngo.via, seven patients showed PR, four patients SD and nine patients PD, respectively. Analyses based on the FIJI software yielded seven patients with PR, four patients with SD and nine patients PD, regarding ΔPSMA-TV and ΔTL-PSMA, respectively.

In terms of agreement between ΔPSMA-TV, ΔTL-PSMA and BR, there were seven patients with a disagreement—in six patients, the discrepancy appeared relevant (non-PD vs. PD classification). These six patients are further characterised in Table 5. 

Of note, a PD classification according to BR was associated with death during follow-up in three of four patients (#4, #8, #18)—despite a reduction of PSMA-TV in all three cases. In contrast, both patients classified PD solely based on ΔPSMA-TV (#10, #15) survived post-therapeutic follow-up period.

## 4. Discussion

PET/CT-derived response criteria are promising biomarkers for monitoring cancer therapies. Among them, FDG PET/CT-derived PERCIST criteria were established first [2]. In the following, researchers evaluated metabolic tumour volume (MTV) and total lesion glycolysis (TLG) from FDG PET/CT scans for their prognostic potential in entities such as cervical or pancreatic cancer [3,6], thereby, serving as an intuitive measure and a medium for quantifying the whole-body tumour burden. 

Paying tribute to the growing importance of PSMA PET/CT imaging, PET/CT-derived criteria were transferred accordingly to PCa patient subgroups. For ^68^Ga-PSMA PET/CT scans, adapted PERCIST criteria performed better than morphological criteria such as RECIST in metastatic PCa [21], ^177^Lu-PSMA-therapy [22] or patients undergoing docetaxel therapy [17]. Schmuck et al. were the first to analyse the overlap of PSMA-TV, TL-PSMA and BR for PCa patients [10]. However, there are scarce data regarding metastatic PCa patients with a high tumour burden—especially patients on taxane-based chemotherapy. 

Going one step back, it is also unknown whether choosing different software solutions for determining metabolic tumour volume influences outcome. For calculation of PSMA-TV and TL-PSMA, different authors used different software (Syngo.via, METAVOL, LifeX or custom-made programs) and currently it is unclear whether their results are seamlessly comparable [11,12,13,14]. To address these issues, we analysed pre- and post-therapeutic ^68^Ga-PSMA PET/CT scans of PCa patients receiving chemotherapy – first, with Syngo.via and afterwards with the FIJI algorithm.

### 4.1. Statistical Agreement of PSMA-TV and TL-PSMA Determined with Syngo.via and FIJI

After determining PSMA-TV and TL-PSMA with both software solutions, statistical analysis revealed no significant differences between the aggregate amounts of PSMA-TV or TL-PSMA determined by Syngo.via or FIJI—thereby indicating that there is no systematic bias or shift caused by one of the software solutions. Next, we compared relative differences between Syngo.via and FIJI for each patient at both time points and used Bland–Altman diagrams for illustration and further calculation. In line with our previous findings, we found a strong statistical comparability for 20 of the 21 CRPC patients examined. In contrast to our results, Kanoun et al. presented divergent results when comparing FDG PET/CTs examined with FIJI and another commercial software application. However, these differences did not have a significant impact on the prognosis of Hodgkin’s lymphoma patients. Moreover, relative changes did not differ significantly [23].

### 4.2. Clinical Agreement of PSMA-TV and TL-PSMA Determined with Syngo.via and FIJI

We then examined PSMA-TV and TL-PSMA determined by Syngo.via and FIJI as imaging-derived biomarkers—starting with Spearman rank correlations with serum PSA levels at baseline and follow-up. We were able to show significant correlations between serum PSA values and PSMA-TV as well as TL-PSMA at baseline and follow-up in accordance to previous studies [10,11,14,24]. These earlier publications showed correlations between relative ΔPSMA-TV and ΔPSMA-TL with relative changes in serum PSA-levels in patients undergoing androgen deprivation, radiotherapy and radioligand therapy [10,12,24]. To our knowledge, our data are the first revealing significant correlations of ΔPSMA-TV, ΔTL-PSMA and ΔPSA for a cohort of patients undergoing taxane-based chemotherapy.

In a patient-wise approach, we compared ΔPSMA-TV and ΔTL-PSMA with the common clinical endpoints BR and PERCIST. Therapy-induced changes in PSMA-TV and TL-PSMA were arbitrarily classified as PR or PD, when a reduction of ≥30% or an increase of ≥30% occurred. Of note, classifying patients’ therapy response according to ΔPSMA-TV and ΔTL-PSMA led to identical results independent of the software applied.

In general, patient subgroups with contradictory results across clinical endpoints—such as BR, PERCIST and PSMA-TV as well as TL-PSMA—surely merit further investigation. Specifically, some patients were classified PD according to PERCIST criteria, while classification according to PSMA-TV and TL-PSMA yielded a SD or PR rating. As described above, classification according to PERCIST was derived from FDG PET/CT and is not yet established for PSMA PET/CT. While it is mainly based on changes in SUVmax, any new lesion leads to a PD rating. In our cohort, there were ten patients with SD or PR according to SUVmax values, but they were rated PD because of new lesions. Regarding this definition, the apparent discrepancy between PERCIST and other PET parameters is put into perspective. Further research on this topic is needed, as an in-depth analysis of the prognostic impact of PERCIST on the overall survival is limited due to our small sample size and was not the primary scope of our study. Brito et al. for example published an approach where they suggested using TL-PSMA for separating PCa patients into two groups with a high or low tumour burden instead of only counting the number of lesions [11].

Within our study, we also observed six patients with a clinically relevant disagreement between PSMA-TV (Syngo.via) and BR. We had a closer look at the follow-up of these patients. While PD classification due to a rise in PSMA-TV was not associated with death in the post-therapeutic follow-up period, a PD status due to BR was associated with three of four patients deceased during follow-up—indicating that PSMA-TV cannot outperform BR as the current gold standard for therapy monitoring of CRPC patients. Of course, more research is needed to thoroughly address this question.

Finally, we want to summarize some important limitations of our study. First, treatment and follow-up were not completely standardized due to the retrospective study design. Second, the sample size of our study is relatively small, but we focused on a clinically homogenous group (metastasized CRPC patient receiving taxane-based chemotherapy) rather than on a high sample size. Third, as segmentation was carried out by two researchers and decisions were made by consensus, we cannot show data for interrater reliability. Although this was not the primary aim of this study, this topic surely merits further research efforts. Fourth, we only picked out two software tools from a larger pool of different platforms. However, we think that choosing a freeware solution for comparison is an attractive approach—as other researchers could compare this application with their commercial in-house solutions.

## 5. Conclusions

Due to the high levels of statistical and clinical agreement in our study cohort of CRPC patients undergoing taxane-based chemotherapy, comparing PSMA-TV and TL-PSMA determined by Syngo.via and FIJI appears feasible. Accordingly, comparing PSMA-TV and TL-PSMA between different departments, e.g., in large multi-centre studies, seems safe. 

In our cohort, prognostic potential of PSMA-TV was not superior to BR.

## Figures and Tables

**Table 1:** Study cohort of *n* = 21 patients receiving chemotherapy for castration-resistant prostate cancer (CRPC). PSMA: prostate-specific membrane antigen; PSA: prostate-specific antigen.

Clinical Characteristics	*n* = 21
**age** [years]	
median (range)	72 (55–93)
**previous therapies** [*n*]	
radical prostatectomy	11 (52%)
radiotherapy	13 (62%)
androgen deprivation therapy	21 (100%)
brachytherapy	1 (5%)
Abiraterone	10 (48%)
Alpharadin	2 (10%)
^177^Lu-PSMA	2 (10%)
**Chemotherapy**	
docetaxel [*n*]	15 (71%)
cycles; median (range)	6 (3–13)
<6 cycles [*n*]	7 (32%)
cabazitaxel [*n*]	7 (32%)
cycles; median (range)	4 (2–8)
**serum PSA baseline** [ng/mL]	
median (range)	15.0 (0–800)
**Gleason score**	
median (range)	8 (6–10)
**metastases localization** [*n*]	
bone	16 (76%)
lymph nodes	18 (86%)
liver	4 (19%)
lungs	2 (10%)
**local recurrence** [*n*]	4 (19%)
**time between baseline PET/CT and PSA** [d]	
median (range)	13 (1–59)
**time between follow-up PET/CT and PSA** [d]	
median (range)	12 (0–52)
**time between end of chemotherapy and PET/CT** [d]	
median (range)	37 (11–120)

**Table 2:** Biochemical and PET/CT-derived clinical response criteria applied within our study. BR: biochemical response; PSA: prostate-specific antigen; PSMA: prostate-specific membrane antigen; SUVmax: maximum standardized uptake value. PSA, SUVmax, PSMA-derived tumour volume (PSMA-TV) and total lesion-PSMA (TL-PSMA) represent follow up values as percentage of baseline values.

	Complete Response CR	Partial Response PR	Stable Disease SD	Progressive Disease PD
**BR**	PSA negative	PSA ≤ 50%	50% < PSA < 125%	PSA ≥ 125%
**PERCIST**	no malignant PSMA uptake	SUV_max_ ≤ 70%	70% < SUV_max_ < 130%	SUV_max_ ≥ 130% or new PSMA-lesion
**PSMA-TV**	no measurable PSMA-TV	PSMA-TV ≤ 70%	70% < PSMA-TV < 130%	PSMA-TV ≥ 130%
**TL-PSMA**	no measurable TL-PSMA	TL-PSMA ≤ 70%	70% < TL-PSMA < 130%	TL-PSMA ≥ 130%

**Table 3:** Spearman rank correlation coefficients of PSMA-TV/TL-PSMA values (baseline, follow-up, relative change) and corresponding serum prostate-specific antigen (PSA) levels—according to the software solution applied. PSMA: prostate-specific membrane antigen; PSMA-TV: PSMA tumour volume; TL-PSMA: total lesion PSMA.

		PSA Baseline	PSA Follow-Up	rel. Δ PSA [%]
**Syngo.via**				
	PSMA-TV baseline	**0.63 (*p* < 0.01)**		
	PSMA-TV follow-up		**0.84 (*p* < 0.01)**	
	ΔPSMA-TV [%]			**0.70 (*p* < 0.01)**
	TL-PSMA baseline	**0.55 (*p* = 0.019)**		
	TL-PSMA follow-up		**0.80 (*p* < 0.01)**	
	ΔTL-PSMA [%]			**0.67 (*p* < 0.01)**
**FIJI**				
	PSMA-TV baseline	**0.64 (*p* < 0.01)**		
	PSMA-TV follow-up		**0.86 (*p* < 0.01)**	
	ΔPSMA-TV [%]			**0.67 (*p* < 0.01)**
	TL-PSMA baseline	**0.56 (*p* = 0.016)**		
	TL-PSMA follow-up		**0.84 (*p* < 0.01)**	
	ΔTL-PSMA [%]			**0.69 (*p* < 0.01)**

**Table 4:** Clinical response in terms of PERCIST criteria, biochemical response (BR), ΔPSMA-TV and ΔTL-PSMA for castration-resistant prostate cancer (CRPC) patients calculated with the Syngo.via and the FIJI software. Patients with ^1^ were rated PD in PERCIST due to new lesions. For patient #14, changes in PSMA-TV and TL-PSMA were not calculated due to tracer extravasation at baseline PET/CT—classification according to PERCIST was applicable due to a new lesion at follow-up. PR: partial response; SD: stable disease; PD: progressive disease.

Patient	PERCIST	BR	ΔPSMA-TV	ΔTL-PSMA	ΔPSMA-TV	ΔTL-PSMA
			Syngo.via	Syngo.via	FIJI	FIJI
#1	**SD**	**SD**	**PR**	**PR**	**PR**	**PR**
#2	**PD ^1^**		**PD**	**PD**	**PD**	**PD**
#3	**PR**	**PR**	**PR**	**PR**	**PR**	**PR**
#4	**PD ^1^**	**PD**	**PR**	**PR**	**PR**	**PR**
#5	**PR**	**PR**	**PR**	**PR**	**PR**	**PR**
#6	**PD ^1^**	**PD**	**PD**	**PD**	**PD**	**PD**
#7	**PR**	**PR**	**PR**	**PR**	**PR**	**PR**
#8	**PD ^1^**	**PD**	**SD**	**SD**	**SD**	**SD**
#9	**PD**	**SD**	**SD**	**SD**	**SD**	**SD**
#10	**PD**	**SD**	**PD**	**PD**	**PD**	**PD**
#11	**PD**	**PD**	**PD**	**PD**	**PD**	**PD**
#12	**PD ^1^**	**PD**	**PD**	**PD**	**PD**	**PD**
#13	**PD ^1^**	**PD**	**PD**	**PD**	**PD**	**PD**
#14	**PD ^1^**	**PD**				
#15	**PD**	**PR**	**PD**	**PD**	**PD**	**PD**
#16	**PD ^1^**	**PR**	**PR**	**PR**	**PR**	**PR**
#17	**PD ^1^**		**PR**	**PR**	**PR**	**PR**
#18	**PR**	**PD**	**SD**	**SD**	**SD**	**SD**
#19	**PD ^1^**	**PD**	**PD**	**PD**	**PD**	**PD**
#20	**PD**	**PD**	**PD**	**PD**	**PD**	**PD**
#21	**PD ^1^**	**PD**	**SD**	**SD**	**SD**	**SD**

**Table 5:** Relative changes in PSA, ΔPSMA-TV (Syngo.via) and overall survival (OS) in six patients with clinically relevant disagreement between PSMA-TV and BR (non-PD vs. PD classification). PR: partial response; SD: stable disease; PD: progressive disease.

Patient	BR	ΔPSMA-TV	ΔPSA [%]	ΔPSMA-TV [%]	OS [d]	Death
#4	**PD**	**PR**	+238%	−76%	216	Yes
#8	**PD**	**SD**	+786%	−21%	235	Yes
#10	**SD**	**PD**	+9%	+42%	1169	No
#15	**PR**	**PD**	−99%	+213%	720	No
#18	**PD**	**SD**	+291%	−14%	328	Yes
#21	**PD**	**SD**	+66%	+26%	1556	No

## Supplementary Materials

The following are available online at https://www.mdpi.com/2077-0383/9/5/1390/s1, Table S1: raw data regarding PSMA-TV and TL-PSMA counts for each patient of our study cohort.
