# Supplementary material for: Metabolic Tumour Volume from PSMA PET/CT Scans of Prostate Cancer Patients during Chemotherapy—Do Different Software Solutions Deliver Comparable Results?
Source: J Clin Med. 2020 May 8;9(5):1390. doi: 10.3390/jcm9051390 (PMC7290891; doi:10.3390/jcm9051390)
Supplement: Supplementary file 1 [file jcm-09-01390-s001.pdf]

Table S1: PSA-values and PET-derived parameters PSMA-TV and TL-PSMA

#	Baseline					Follow-up				
	PSA	PSMA-TV Syngo.via	TL-PSMA Syngo.via	PSMA-TV FIJI	TL-PSMA FIJI	PSA	PSMA-TV Syngo.via	TL-PSMA Syngo.via	PSMA-TV FIJI	TL-PSMA FIJI
1	1,9	1,08	9,78	1,08	9,78	2	0,17	1,47	0,17	1,47
2	0,8	53,74	1392,67	49,93	1357,2	neg.	99,53	2192,72	116,03	2605,09
3	23,43	8,79	81,36	9,62	88,09	0,41	0,25	1,76	0,33	2,28
4	195	323,05	2402,92	348,93	2533,54	659	77,30	713,35	76,47	697,12
5	1,49	1,66	12,71	4,31	40,72	0,01	0,08	0,56	0,08	0,56
6	47,4	131,21	1443,22	132,7	1457,82	151,3	378,70	3182,02	357,22	3187,08
7	20,5	7,22	97,13	7,38	98,55	0,5	0,17	1,24	0,17	1,24
8	15	88,93	555,95	90,57	556,86	132,9	70,58	561,58	75,14	609,89
9	0,544	58,39	736,66	60,71	759,82	0,536	44,54	596,20	43,13	579,92
10	3,66	9,46	76,14	8,96	71,84	4	13,44	121,8	13,1	117,75
11	8,24	13,27	69,84	13,27	69,88	1997	830,14	4223,00	1471,35	9576,02
12	35,3	119,93	2108,07	130,46	2271,32	176,1	930,42	11725,49	616,49	9005,13
13	151,46	255,7	2978,61	263,5	3060,47	1293,71	820,69	9185,73	627,69	7235,86
14	800	extravasate	extravasate	extravasate	extravasate	3068,7	1609,61	8543,65	1774,08	10641,35
15	1,77	2,99	32,45	2,41	26,59	0,01	9,37	110,66	8,46	101,16
16	38	2,49	9,2	3,15	11,53	10,49	0,08	0,61	0,08	0,61
17	0	112,05	1073,92	110,48	1057,27	neg.	70,83	663,39	67,26	626,27
18	3,4	3,07	38,64	3,07	38,64	13,3	2,65	27,92	2,57	27,25
19	79,6	29,61	256,6	28,61	266,35	717	248,4	1414,58	277,77	1701,69
20	60,49	139,84	1973,37	130,38	1901,83	334,2	960,69	12439,05	658,62	10817,67
21	14,9	181,64	3597,01	167,46	3445,01	24,7	228,91	2756,75	198,31	2419,89
